# West Nile virus in humans, Greece, 2018: the largest seasonal number of cases, 9 years after its emergence in the country

**DOI:** 10.2807/1560-7917.ES.2020.25.32.1900543

**Published:** 2020-08-13

**Authors:** Danai Pervanidou, Annita Vakali, Theano Georgakopoulou, Takis Panagiotopoulos, Eleni Patsoula, George Koliopoulos, Constantina Politis, Kostas Stamoulis, Elpida Gavana, Styliani Pappa, Maria Mavrouli, Maria Emmanouil, George Sourvinos, Andreas Mentis, Athanassios Tsakris, Christos Hadjichristodoulou, Sotirios Tsiodras, Anna Papa

**Affiliations:** 1 Hellenic National Public Health Organization/former Hellenic Center for Disease Control & Prevention, Athens, Greece; 2 School of Public Health, Faculty of Public Health Policy, University of West Attica, Athens, Greece; 3 Benaki Phytopathological Institute, Athens, Greece; 4 Hellenic National Blood Transfusion Center, Athens, Greece; 5 National Reference Center for Arboviruses and Haemorrhagic Fever Viruses, Department of Microbiology, Medical School, Aristotle University of Thessaloniki, Thessaloniki, Greece; 6 Department of Microbiology, Medical School, National and Kapodistrian University of Athens, Athens, Greece; 7 Diagnostic Services Laboratory, Public Health Laboratories, Hellenic Pasteur Institute, Athens, Greece; 8 Laboratory of Clinical Virology, Medical School, University of Crete, Heraklion, Crete, Greece; 9 Laboratory of Hygiene and Epidemiology, Medical School, University of Thessaly, Larisa, Greece; 10 National and Kapodistrian University of Athens, Athens, Greece

**Keywords:** West Nile Virus, Greece, vectorborne diseases, 2018

## Abstract

**Background:**

Human cases of West Nile virus (WNV) infection are recorded since 2010 in Greece, with seasonal outbreaks occurring almost annually. Enhanced surveillance has been implemented since 2010, to promptly characterise cases’ temporal and geographical distribution and inform authorities for implementation of appropriate measures (mosquito control, health education, blood safety).

**Aim:**

We describe the epidemiology of WNV human infections in Greece focusing on the 2018 season.

**Methods:**

The National Public Health Organization advised physicians to test all suspect WNV infection cases and refer samples to reference laboratories. Laboratories notified diagnosed cases on a daily basis. Treating physicians, patients, and infected blood donors were interviewed within 48 hours after diagnosis and the probable infection location was identified. Hospitalised cases were followed up until discharge.

**Results:**

A total of 317 autochthonous WNV infection cases were diagnosed in 2018. Among them, 243 cases had neuroinvasive disease (WNND), representing a 23% increase of WNND cases compared with 2010, the previous most intense season. There were 51 deaths. Cases started occurring from week 22, earlier than usual. Both rural and urban areas were affected, with 86 (26% of the total) municipalities belonging to seven (54% of the total) regions recording cases. Two major epicentres were identified in Attica and Central Macedonia regions.

**Conclusions:**

The largest number of human cases of WNV infection ever recorded in Greece occurred in 2018, with a wide geographical distribution, suggesting intense virus circulation. Enhanced surveillance is vital for the early detection of human cases and the prompt implementation of response measures.

## Introduction

West Nile virus (WNV) is a flavivirus primarily transmitted to humans, equids and other mammals through the bites of infected mosquitoes, mainly of the *Culex* genus [1]. WNV lineages 1 and 2 have been associated with significant outbreaks in humans [1]. Birds serve as reservoir hosts [1], whereas humans and equids are considered dead-end hosts [2-4]. Transmission through blood transfusion and organ transplantation can occasionally occur, and other rare modes of transmission have been also recorded, such as transmission from mother to child during pregnancy, delivery, or breastfeeding, and in laboratory settings [1,3].

Most humans infected with WNV remain asymptomatic; ca 20% develop a disease with fever and/or other influenza-like symptoms known as West Nile fever (WNF), and less than 1% develop neuroinvasive disease (WNND), such as encephalitis, meningitis or – more rarely – acute flaccid paralysis (AFP) [5,6]. Elderly and immunocompromised persons are at higher risk of developing severe disease and having a fatal outcome [7]. No specific treatment or vaccine exists to cure or prevent the disease in humans.

WNV infection is considered endemo-epidemic in parts of Europe, affecting countries in southern, eastern and western Europe [8,9] and is considered a re-emerging public health challenge in the European Union (EU), with annual seasonal outbreaks during the summer months and early autumn.

Migratory birds are thought to be the source of introductions of new virus strains into previously unaffected areas [4,10-15]. In Europe the virus has been introduced through migratory birds from Africa [16,17] and its circulation is probably greatly influenced by the flying routes of migratory bird species [18]. WNV overwinters in mosquitoes [19], and overwintering in local bird species in Europe cannot be excluded [8,17]. WNV lineage 1 was the main lineage circulating in Europe [10,20] from the 1960s, whereas lineage 2 spread over central and southern eastern Europe [21,22] since 2004 causing major outbreaks [23-27]. In 2018, the largest number of cases of WNV infection was recorded in Europe, with their total number exceeding the cumulative number of all cases recorded in the previous 7 years, between 2010 and 2017 [28].

In Greece, human cases of WNV infections have been recorded since 2010 [29,30], with seasonal outbreaks (from end June to early October) on an almost annual basis [31-35]. The causative WNV strain (Nea Santa-Greece-2010) belongs to the Central European/Hungarian subclade of WNV lineage 2 [25,36,37]. This strain was detected in patients, blood donors, mosquitoes, horses and birds in many seasons from 2010 to 2017 [25,35,38-47].

Enhanced surveillance of human WNV infection is annually implemented since 2010, from May to November, by the Hellenic National Public Health Organization (NPHO)/former Hellenic Center for Disease Control & Prevention (HCDCP). The surveillance is undertaken to promptly identify human cases of WNV infection and monitor their temporal and geographical distribution. It also has the objective to detect cases and WNV-affected areas, in a timely fashion, to inform national and local authorities for the implementation of appropriate response measures, including blood safety measures, intensified vector control and communication campaigns. In the long term, surveillance aims to quantify the disease burden, and identify seasonal, geographical and demographic patterns, and populations at risk [31]. Herein, we describe the clinical characteristics and laboratory findings of human cases as well as their distribution in time and place during 2018 in Greece.

## Methods

### Surveillance

As part of routine procedures implemented over the last years before the beginning of each WNV season, informative material was sent by the NPHO/former HCDCP to all healthcare facilities and medical associations around the country in May 2018, to raise awareness of physicians concerning the diagnosis of WNV infection. The NPHO/former HCDCP prompted physicians to further conduct laboratory testing of all WNV infection suspected cases, who were defined as any person with acute onset of neurological syndromes (encephalitis, meningitis or myelitis), as well as any person with non-neurological illness but unexplained fever. NPHO/former HCDCP recommended referral of samples to specific specialised laboratories for testing.

The Vector-borne Diseases (VBD) Office of the NPHO/former HCDCP implemented active laboratory-based surveillance, throughout the transmission season, with daily communication with three reference/specialised laboratories (further described in the ‘laboratory methods’ section) and daily reporting of diagnosed cases to NPHO/former HCDCP. Laboratory diagnosis was free-of-charge for patients in the reference/specialised laboratories, reimbursed by NPHO/former HCDCP.

Blood safety authorities informed NPHO/former HCDCP about any infections diagnosed among blood donors tested in affected areas.

The EU case definition of WNV infection [48] was used with slight modifications, in that only laboratory – and not epidemiological – criteria were used to define probable cases; a confirmed case was defined as a person with either PCR detection of WNV nucleic acid in any biological specimen, such as blood, cerebrospinal fluid (CSF) or urine, or detection of a WNV specific IgM antibody response in CSF, or WNV IgM and IgG antibody response and confirmation by neutralisation tests. A probable case was defined as a person with positive IgM antibody response only in serum. Asymptomatic infections detected during blood screening in affected areas were also included – for the first time in 2018 – in the case definition.

Municipalities (the lowest administrative unit) where at least one human laboratory-diagnosed case of WNV infection was exposed during the 2018 period were defined as affected areas.

### Data collection

The VBD Office of the NPHO/former HCDCP investigated and recorded all cases with laboratory-diagnosed WNV infection, symptomatic or not, with or without neuroinvasive disease. The disease was classified as WNND (encephalitis, meningitis, meningo-encephalitis or myelitis/AFP) based on the treating physicians’ clinical assessment and/or additional laboratory and/or imaging findings, when available.

Cases were investigated by the VBD Office with in-depth telephone interviews with the patients, or if this was not possible, with their close relatives, ideally within 24–48 hours after diagnosis, using a standardised investigation form, to obtain detailed travel history during incubation period (2–14 days before symptom onset) and identify the probable place of exposure. In case of complex travel history, the most probable place of infection was defined – after risk assessment – by a multi-sectoral working group of the Ministry of Health dedicated to designate the areas affected by VBD.

The treating physician was interviewed to record the clinical manifestations and underlying diseases of each patient.

The data collected included: demographic characteristics, clinical manifestations, underlying chronic diseases, dates of onset of symptoms, hospitalisation, diagnosis, discharge, admission and discharge from intensive care unit (ICU), potential risk factors, laboratory results, detailed travel history during the maximum incubation period. Hospitalised patients were actively followed up on a daily basis, by telephone, and their outcome at the end of hospitalisation was recorded.

The NPHO/former HCDCP informed on a daily basis, by e-mails, national, regional and local public health authorities about the cases and their probable place of exposure, in order to implement responsive preventive measures (vector control, communication activities, blood safety).

Weekly surveillance reports were published on the NPHO/former HCDCP website.

### Laboratory methods

On a daily basis, the VBD Office of the NPHO/former HCDCP received laboratory data of the diagnosed cases mainly from three laboratories (including the National Reference Center for Arboviruses and Haemorrhagic Fever Viruses, Department of Microbiology, Medical School, Aristotle University of Thessaloniki, the Department of Microbiology, Medical School, National and Kapodistrian University of Athens and the Diagnostic Services Laboratory, Public Health Laboratories, Hellenic Pasteur Institute of Athens) in which the vast majority of suspected cases were tested and 99% of cases were diagnosed in 2018.

 Serum and CSF specimens were tested for the presence of WNV-specific IgM and IgG antibodies using commercial ELISA kits (Focus Technologies, Cypress, CA, United States). Reverse transcription (RT)-PCR tests (in house and commercial) were performed on RNA extracted from blood, CSF or urine samples. The National Reference Center for Arboviruses performed an RT-nested PCR, which amplifies a 520-bp fragment of the WNV non-structural protein 3 (NS3) gene on PCR-positive samples [38]. All PCR products were sequenced in a 3130 ABI Genetic Analyzer (Applied Biosystems, Foster City, CA, US). Nucleotide sequences were aligned with similar sequences of the same genomic region retrieved from the GenBank database, and phylogenetic analysis was performed using the molecular evolutionary genetics analysis (MEGA)7 software [49]. The National Reference Center for Arboviruses performed also neutralisation tests in PCR-negative, IgG-positive serum samples and applied PCR combined with Sanger sequencing for the genetic characterisation of the strain(s); in addition, next generation sequencing was performed on selected samples.

### Blood safety measures

Blood safety measures for the protection of blood donations against WNV infection were implemented nationwide for blood donors residing or having visited affected municipalities. These measures included blood donor deferral or screening of donated blood for WNV RNA, with targeted individual donation (ID) nucleic acid amplification testing (NAT), and haemovigilance (i.e. surveillance of serious adverse or unexpected events or reactions in donors or recipients, epidemiological follow-up of donors, as referred to in Article 4 and Annex III of the Commission Directive 2004/33/EC [50]).

### Data analysis

We performed descriptive analysis of the surveillance data, i.e. geographical and temporal distribution of human cases with WNV infection, demographic characteristics (age, sex), clinical manifestations, underlying diseases and clinical outcome.

We assigned week numbers using the International Organization for Standardization (ISO) 8601 standard [51].

### Ethical statement

No ethical approval was needed for this study, as no individual data were identifiable, and only aggregated data were analysed and presented.

## Results

On week 26/2018, NPHO/former HCDCP was notified of the first six human WNV infection cases in the season, in Attica Region, with symptom onset on 31 May (week 22), and within the first fortnight of June. Cases continued to occur throughout the 2018 transmission period, in several areas of the country.

A total of 317 autochthonous cases of WNV infection were recorded in 2018 all over Greece, including 243 WNND cases, 68 WNF cases, and six asymptomatic cases (blood donors). The overall WNND incidence was 2.25 cases per 100,000 population; this was the highest incidence ever recorded in Greece, with a 23% statistical significant increase compared with 2010 (p = 0.03), the previous most intense season (Table 1). Two of the 317 reported WNV infection cases were diagnosed abroad, in Czech Republic and in Italy respectively. One more WNND case was classified as imported from Romania (not included in the current analysis).

**Table 1:** Numbers of total WNV affected areas^a^, infection cases, WNND cases and related deaths and case fatality per year, as well as annual incidence of total WNND cases, Greece, 2010–2018^b^ (n = 989 WNV infection cases)

Criteria	2010	2011	2012	2013	2014	2015	2016	2017	2018^b^
Number of affected municipalities	38	46	42	35	7	0	0	10	86
Number of affected regional units^c^	11	21	19	12	4	0	0	6	24
Number of affected regions	5	7	8	5	3	0	0	3	7
Number of WNV infection cases	262	100	161	86	15	0	0	48	317
Number of WNND cases	197	75	109	51	14	0	0	28	243
Percentage of WNND cases	75%	75%	68%	59%	93%	NA	NA	58%	77%
Incidence of WNND cases (per 100,000 population)	1.8	0.7	1.0	0.5	0.1	0	0	0.3	2.2
Number of fatal cases with WNV infection	35	9	18	11	6	0	0	5	51
Case fatality among cases with WNV infection	13%	9%	11%	13%	40%	NA	NA	10%	16%
Number of fatal cases with WNND	33	9	18	10	6	0	0	5	48
Case fatality of cases with WNND	17%	12%	17%	20%	43%	NA	NA	18%	20%

NA: not applicable; NUTS: nomenclature of territorial units for statistics; WNND: West Nile virus neuroinvasive disease; WNV: West Nile virus.

^a^ Number of affected municipalities, regional units^b^, and regions with ≥ 1 case of WNV infection.

^b^ To put the 2018 data in a further more recent perspective, the numbers for 2019 were: 56 affected municipalities, 19 affected regional units, five affected regions, 227 WNV infection cases, 140 WNND cases (corresponding to 62% of WNV infection cases), 1.3 WNND cases per 100,000 population, 35 fatal cases with WNV infection (corresponding to a case fatality of 15% among cases with WNV infection) and 33 fatal cases with WNND (representing a case fatality of 24% in cases with WNND).

^c^ Regional units: NUTS3.

Eleven cases of WNV infection were blood donors (aged 30–62 years old) diagnosed in affected areas; five of these cases developed symptoms and six remained asymptomatic.

In 2018, 77% of cases presented with WNND, compared with a total of 71% (474/672) of cases diagnosed in the previous 2010–2017 period, ranging from 58% in 2017 to 93% in 2014 (Table 1).

A total of 312 (98%) cases of WNV infection (or their close relatives) were directly interviewed by NPHO/former HCDCP. These included the exported case diagnosed in Italy, who was also investigated in collaboration with the Italian public health authorities. Four cases (or their relatives) diagnosed in Greece could not be directly contacted, and the exported case diagnosed in the Czech Republic was investigated by the Czech public health authorities. Close relatives of 235 (75%) cases were interviewed.

For 10 cases of the 312 interviewed cases, the exact date of diagnosis in the laboratory was missing. For the remaining cases, these were investigated within a median period of 1 day (range: 0–29) after diagnosis; 74% (222/302) of cases were interviewed within 2 days after diagnosis, and 61% (183/302) within 1 day.

All symptomatic cases (n = 311) had symptom onset within the 5 month period from 31 May 2018 (week 22/2018) to 29 October 2018 (week 44/2018), while the last date of positive blood sample was on 2 November 2018 from an asymptomatic blood donor. The number of recorded WNND cases peaked in weeks 31 and 32 (with 32 and 33 WNND cases per week, respectively).

**Figure 1:**
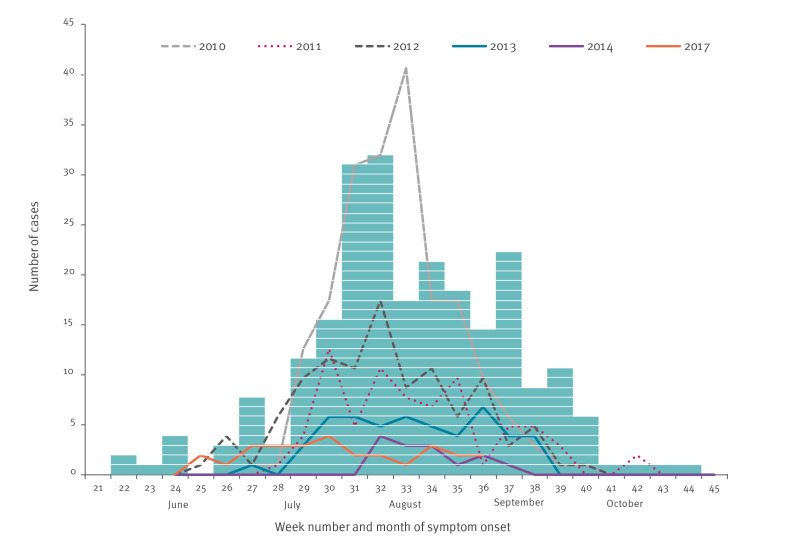
Number of laboratory diagnosed West Nile virus neuroinvasive disease cases by week of symptom onset, Greece, 2010–2018^a^ (n = 242)^b^

^a^ In 2015 and 2016 no cases were recorded. Each box represents one WNND case diagnosed and reported in 2018.

^b^ One WNND case with undetermined week of symptom onset is not included.

Figure 1 shows the reported WNND cases by week of symptom onset in 2018, compared with previous seasons (2010–2017).

For three cases, the probable place of exposure could not be determined. The remaining WNV infection cases were exposed in a total of 86/325 (26%) municipalities, 24/74 (32%) regional units, and 7/13 (54%) regions, indicating a wide geographical distribution of cases compared with previous transmission periods (Table 1).

**Figure 2:**
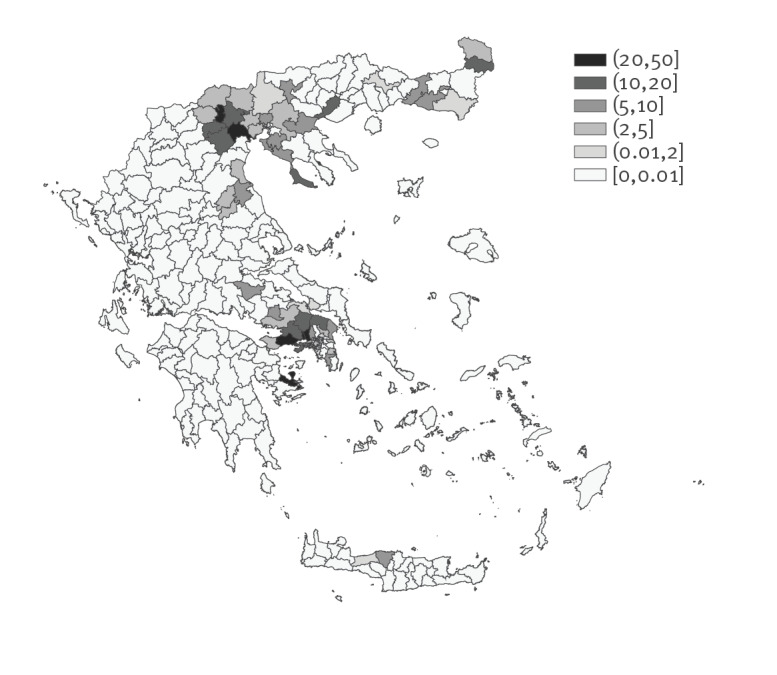
Incidence (per 100,000 population) of West Nile virus neuroinvasive disease by probable municipality of exposure, Greece, 2018 (n = 242)^a^

^a^ For one case, the suspected municipality of exposure could not be determined.

The geographical distribution of WNND cases is presented in Figure 2. Two major epicentres were recorded, in the regions of Attica and Central Macedonia (Table 2, Figure 2), with the highest number of WNV infection and WNND cases ever recorded in Attica and the second highest number of cases ever recorded in Central Macedonia (since 2010) (Table 2).

**Table 2:** Number of WNV infection cases and WNND cases per probable region of exposure and year, Greece, 2010–2018^a^ (n = 989 WNV infection cases)

Probable region of exposure	Number of recorded WNV infection cases (number of WNND cases) per year^a^						
	2010	2011	2012	2013	2014	2017	2018
Attica	0 (0)	30 (21)	45 (30)	35 (24)	2 (2)	0 (0)	160 (136)
Central Macedonia	250 (186)	31 (21)	20 (15)	21 (13)	0 (0)	0 (0)	117 (78)
West Macedonia	1 (1)	1 (1)	0 (0)	0 (0)	0 (0)	0 (0)	0 (0)
East Macedonia and Thrace	1 (1)	0 (0)	76 (46)	27 (11)	11 (10)	0 (0)	14 (11)
Sterea Ellada	0 (0)	6 (6)	1 (1)	0 (0)	0 (0)	0 (0)	10 (7)
Thessaly	9 (8)	30 (25)	0 (0)	0 (0)	0 (0)	0 (0)	8 (7)
Peloponnese	0 (0)	0 (0)	0 (0)	0 (0)	0 (0)	43 (23)	2 (1)
Crete	0 (0)	0 (0)	0 (0)	0 (0)	0 (0)	1 (1)	2 (2)
West Greece	1 (1)	1 (1)	8 (7)	1 (1)	2 (2)	4 (4)	0 (0)
Ionian islands	0 (0)	0 (0)	4 (4)	1 (1)	0 (0)	0 (0)	0 (0)
North Aegean	0 (0)	0 (0)	2 (2)	0 (0)	0 (0)	0 (0)	0 (0)
South Aegean	0 (0)	1 (0)	0 (0)	0 (0)	0 (0)	0 (0)	0 (0)
Ipeiros	0 (0)	0 (0)	1 (1)	0 (0)	0 (0)	0 (0)	0 (0)
Unknown/undetermined	0 (0)	0 (0)	4 (3)	1 (1)	0 (0)	0 (0)	4 (1)
Total	262 (197)	100 (75)	161 (109)	86 (51)	15 (14)	48 (28)	317 (243)

WNND: West Nile virus neuroinvasive disease; WNV: West Nile virus.

^a^ As no cases of WNV infection in Greece were recorded in 2015 and 2016, these years are not shown in the Table.

Among the 24 regional units (nomenclature of territorial units for statistics (NUTS)3 level) affected in 2018, 22 were affected at least once in previous seasons, and 98% (308/314) of cases occurred in regional units previously affected. Cases occurred both in rural and urban areas, and large cities were also affected, including the capital.

**Figure 3:**
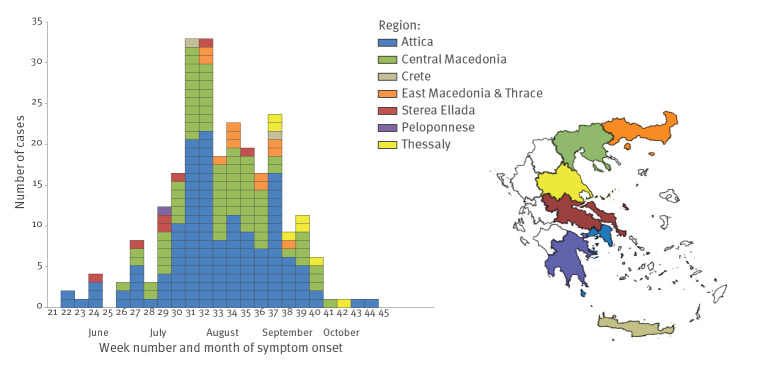
Number of laboratory-diagnosed West Nile virus neuroinvasive disease cases by week of symptom onset and region of exposure, Greece, 2018 (n = 241)^a^

In June 2018, cases were recorded mainly from Attica (and a couple of cases from the nearby Sterea Ellada region). From July, cases were also recorded in Central Macedonia, and from August, in East Macedonia and Thrace region, in north-eastern Greece. In September–October, cases were further recorded in Thessaly region. A couple of cases were also recorded in Crete and Peloponnese regions (Figure 3).

WNND: West Nile virus neuroinvasive disease.

^a^ One WNND case with undetermined week of symptom onset and one WNND case with undetermined region of exposure (with symptom onset at week 32/2018) are not included.

For cases of WNV infection with available information on their place of exposure, the vast majority (87%; 268/309) were infected in their place of permanent residence (the rest, mainly in the place of summer vacation).

The median age of the 243 WNND cases in 2018 was 75 years (range: 10–95 years), significantly higher (p = 0.012) than the median age of the total 474 WNND cases diagnosed in the previous 2010–2017 period (72 years; range: 2–95). Among WNND cases, 81% (196/243) were aged 60 years or older. The incidence of WNND cases increased from 0.14 per 100,000 population in the less than 20 year-olds to 10.66 per 100,000 population in those who were ≥ 80 years old (Table 3). The age of WNND cases in 2018 (median age: 75 years; range: 10–95) was significantly higher (p < 0.001) than that of the diagnosed symptomatic WNF cases (median age: 65 years; range: 19–97).

**Table 3:** Age and sex distribution of West Nile virus neuroinvasive disease cases, Greece, 2018 (n = 243)

Characteristics	Number of cases	Incidence rate (per 100,000 population)^a^	Risk ratio (95% confidence interval)
Age group (in years)			
< 20	3	0.14	Reference
20–29	3	0.27	1.85 (0.37–9.15)
30–39	13	0.92	6.38 (1.82–22.38)
40–49	11	0.68	4.73 (1.32–16.95)
50–59	17	1.15	7.97 (2.34–27.21)
60–69	35	2.76	19.21 (5.91–62.46)
70–79	82	8.28	57.62 (18.21–182.37)
≥80	79	10.66	74.22 (23.43–235.09)
Sex			
Female	86	1.55	Reference
Male	157	3.01	1.94 (1.49–2.52)

^a^ Population data from Hellenic Statistical Authority (EL.STAT.) [63].

Among the 317 WNV infection cases and the 243 WNND cases, 62% (n = 198) and 65% (n = 157) were male, respectively. The WNND incidence among males was almost two times higher than that among females (p < 0.001) (Table 3).

The median period from symptom onset to diagnosis for 239 WNND cases with available relevant information was 11 days (range: 2–45), as well as for 63 non-WNND cases, excluding blood donors (range: 3–57). The median period from admission to hospital to diagnosis for 295 cases of WNV infection was 6 days (range: 0–40).

Among the 243 WNND cases, 206 (85%) had encephalitis/meningoencephalitis (including 11 patients also presenting AFP), 34 (14%) cases had meningitis, and three cases had AFP signs only (Table 4).

**Table 4:** Number and percentage of WNND cases (n = 243) as well as number and percentage of fatal cases with WNND (n = 48), by clinical presentation, Greece, 2018

Parameter	Encephalitis	Meningo -encephalitis	Meningitis	AFP only	AFP and encephalitis/ meningitis
Number of WNND cases	128	78	34	3	11
Percentage of total WNND cases	53%	32%	14%	1%	5%
Number of fatal cases with WNND	31	14	3	0	3
Percentage of fatal cases among total WNND fatal cases	65%	29%	6%	NA	10%

AFP: acute flaccid paralysis; NA: not applicable; WNND: West Nile virus neuroinvasive disease.

In each row where numbers of cases are presented, the sum of the cases in the respective row can be more than the total number of cases, as a given case could have more than one presentation.

A total of 311 (98%) diagnosed WNV infection cases reported clinical symptoms, the most common being fever, followed by malaise/fatigue, confusion/consciousness level deterioration, anorexia, sleepiness, chills, headache, gastrointestinal symptoms (diarrhoea, nausea, vomiting, abdominal pain), dizziness, myalgia/arthralgia, extrapyramidal signs (tremor, parkinsonism), ataxia/gait disorders, rash, limb paralysis, numbness, retro-orbital pain, vision deterioration, lymph nodes enlargement (Table 5).

**Table 5:** Symptoms^a^ of West Nile virus neuroinvasive disease cases (n = 243) and West Nile fever cases (n = 68), Greece, 2018^b^

Symptom	WNND cases		WNF cases	
	Proportion^b^ with the symptom	Percentage	Proportion^b^ with the symptom	Percentage
Fever	240/242	99	65/68	96
Malaise/fatigue	212/231	92	60/67	90
Confusion consciousness level deterioration	179/232	77	13/67	19
Anorexia	139/189	74	44/64	69
Sleepiness	159/219	73	17/62	27
Chills	136/201	68	30/62	48
Headache	129/227	57	42/68	62
At least one gastrointestinal symptom (i.e., diarrhoea, nausea, vomiting, abdominal pain)	134/237	57	39/66	59
Dizziness	103/207	50	18/64	28
Myalgia/arthralgia	94/221	43	30/67	45
Vomiting	90/238	38	20/67	30
Extrapyramidal signs (tremor, parkinsonism)	77/207	37	0/66	NA
Ataxia, gait disorders	47/150	31	0/64	NA
Diarrhoea	50/237	21	18/66	27
Nausea	47/237	20	18/67	27
Rash	39/235	17	15/67	22
Abdominal pain	33/223	15	7/66	11
Limb paralysis	24/213	11	0/66	NA
Numbness	15/155	10	3/57	5
Retro-orbital pain	17/181	9	9/62	15
Vision deterioration	11/183	6	1/64	2
Lymph nodes enlargement	3/66	5	5/32	16

CKP: creatine phosphokinase; NA: not applicable; SIADH: syndrome of inappropriate antidiuretic hormone secretion; WNF: West Nile fever; WNND: West Nile virus neuroinvasive disease.

^a^ Other symptoms/signs reported from the treating physicians included: acute respiratory failure (n = 22), bradypsychismus (n = 16), seizures (n = 15), respiratory infection (n = 13), dysarthria-speech disorders (n = 12), acute renal failure (n = 10), diplopia (n = 9), myoclonous (n = 9), aphasia/apraxia (n = 8), arrhythmia (n = 5), cough (n = 5), hepatitis/liver dysfunction (n = 5), rhabdomyolysis/increased CPK (n = 5), SIADH/hyponatraemia (n = 5), biphasic fever (n = 4), pulmonary oedema (n = 4), sore throat (n = 4), chorea/ ballistic movements (n = 3), dysautonomia (n = 3), hearing disorders (n = 3), myocarditis/increased troponine (n = 3), pulmonary embolism (n = 3), hypokalaemia (n = 2), acute epiglottitis (n = 1), aneurysm rupture (n = 1), blepharoptosis (n = 1), Guillain–Barré syndrome (n = 1), retinitis (n = 1) and uveitis (n = 1),

^b^ Number of cases with the symptom in question/number of cases with known information.

Among WNND cases, 83% (201/242) reported at least one underlying chronic disease, including cardiovascular diseases (62%; 150/242, including stroke and heart disease), heart disease (60%; 144/242, including hypertension), hypertension (45%; 110/242), diabetes mellitus (31%; 75/241), coronary heart disease (19%; 45/242), chronic neuropsychiatric disease (16%; 39/242), arrhythmia (13%; 32/242), cancer (13%; 31/241), stroke (9%; 22/240), valvulopathy/heart failure (7%; 17/242), other immunosuppression than diabetes and cancer (7%; 16/242, including autoimmune disorders, hepatic cirrhosis, organ transplant, myasthenia, corticosteroids treatment), thyroid disease (7%; 18/242), chronic renal failure (6%; 14/242), respiratory disease (4%; 9/242), alcohol abuse (1%; 2/240).

Among the 311 symptomatic cases, 94% (n = 291) were hospitalised: 99% (241/243) of the WNND cases and 74% (50/68) of the symptomatic non-WNND cases.

The median duration of hospitalisation of 239 hospitalised cases of WNV infection discharged from the hospital was 10 days (range: 1–102), while among 192 WNND cases (hospitalised and discharged from the hospital) it was 11 days (range: 2–102). Forty-eight cases were hospitalised in ICU: 47 with WNND, while the one with non-WNND had other co-morbidities. The median duration of hospitalisation in ICU (before discharge or fatal outcome) was 19 days (range: 1–237).

A total of 51 deaths were recorded during hospitalisation, with an overall case fatality (CF) of 16% among all cases diagnosed with WNV infection. Among fatal cases, 48 had WNND, with a 20% CF among cases with WNND; similar with the total CF among cases with WNND observed during the 2010–2017 period (81/474, 17%) (Table 1). The clinical presentation of WNND among fatal cases is described in Table 4. The CF of the encephalitis/meningo-encephalitis cases was 22% (45/206), whereas it was 9% (3/34) among meningitis cases. The median age of fatal cases was 79 years (range: 63–97). The median period from symptom onset to death (during hospitalisation) was 21 days (range: 6–243).

The vast majority of patients (67%; 204/306) were retired, 38% (117/308) reported doing agricultural or gardening activities, and 22% (67/306) reported having a routine outdoor activity after dawn.

National and regional public health authorities were informed about diagnosed cases and their probable place of exposure, within a few hours after their investigation, and within 24 hours after their diagnosis, via confidential emails.

### Laboratory results

Of the 317 cases, 198 (62%) were confirmed, either by WNV-specific IgM antibody response in CSF (n = 124), and/or positive PCR in any sample (n = 77) (in blood, and/or CSF, and/or urine), and/or by WNV-specific IgM and IgG antibody response in serum and confirmation by neutralisation (n = 29). A total of 97 cases were confirmed by IgM antibody response in CSF only, 50 cases were confirmed by positive PCR in any sample only, 20 cases were confirmed by neutralisation only, and 31 cases were confirmed by more than one confirmatory methods.

A total of 119 cases were considered as probable, since the diagnosis was based only on the detection of WNV-specific IgM antibodies in serum.

**Figure 4:**
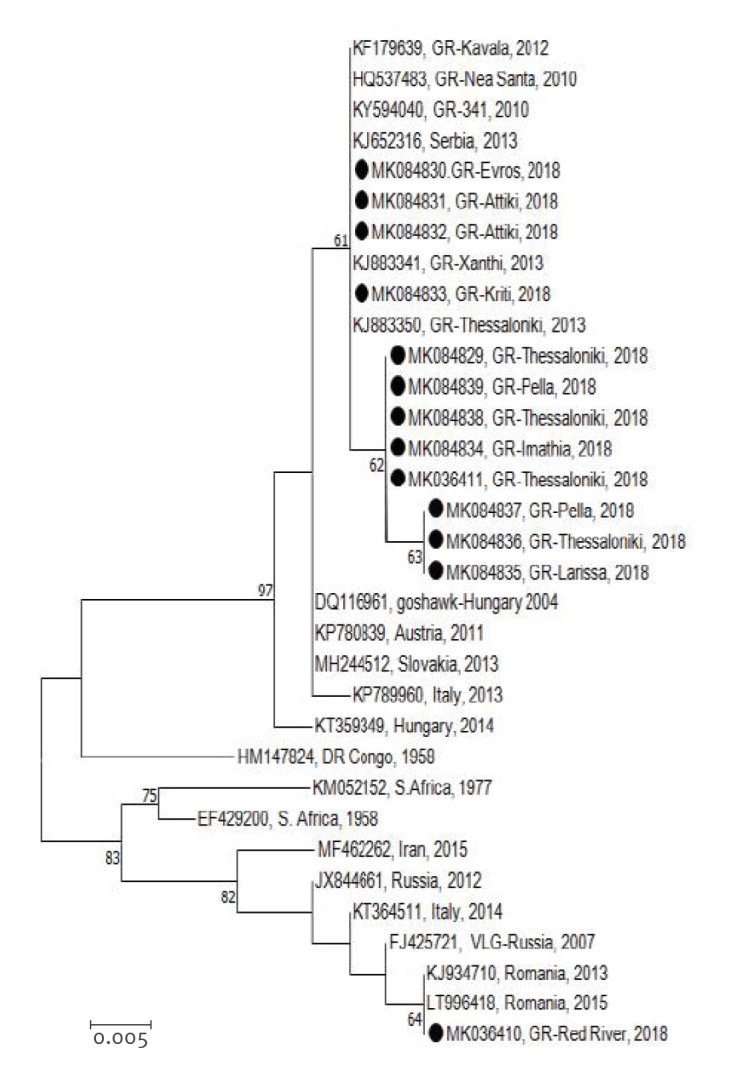
Phylogenetic analysis of West Nile virus strains detected in Greece in 2018

In all but one of the 15 sequenced samples, the sequences clustered into the Central European/Hungarian subclade of WNV lineage 2, similar to the strain of the 2010 outbreak (Nea-Santa-Greece-2010 strain). One sequence taken from a case in Thrace region (northern-eastern Greece) clustered within the Eastern European/Russian subclade of WNV lineage 2 (Figure 4) [52].

NS3: non-structural protein 3.

Neighbour-Joining phylogenetic tree based on 328-bp fragment of West Nile virus NS3 gene. The evolutionary distances were computed using the Kimura 2-parameter method. The tree is drawn to scale, with branch lengths measured in the number of substitutions per site. The percentage of replicate trees in which the associated taxa clustered together in the bootstrap test (1,000 replicates) is shown next to the branches. Bootstrap values lower than 60% are not shown. Sequences in the trees are identifiable by GenBank accession number, country and year of isolation. Sequences of the present study are marked.

While 15 sequences from the 2018 outbreak were obtained, two sequences were slightly shorter than the rest, and they were not included in the phylogenetic tree.

In total, among the cases tested, 99.6% (275/276) had WNV-specific IgM antibodies in serum and 95% (124/131) had WNV-specific IgM antibody response in CSF. In 29 cases with positive IgM and IgG antibodies in serum, the diagnosis was confirmed by neutralisation. A total of 52 cases had positive PCR in blood, 37 cases had positive PCR in urine (in 10 of which this was the method of confirmation), and eight cases had positive PCR in CSF. The median time from symptom onset to sampling for the cases with positive PCR in blood was 6 days (range: -3 to 23), for the cases with positive PCR in urine 6 days (range: 2–20) and for cases with positive PCR in CSF 9 days (range: 1–20).

## Discussion

WNV infections show a strong seasonal pattern in Europe, with the first human cases usually observed in June and most cases recorded from July to October [9]. Accordingly, from 2010 to 2017, human cases of WNV infection in Greece were recorded from the end of June until the beginning of October. In contrast, the onset of symptoms in the first recorded human case in 2018 was in week 22 (end of May), making this the earliest WNV transmission season ever noted in the country and in Europe. The seasonal outbreak moreover lasted until early November (Figure 1) and coincided with the longest season (i.e. more than 5 months) in the last years in Europe [9], suggesting a possible need to widen the surveillance period.

The number of recorded WNND cases in Greece in 2018 peaked in weeks 31 and 32 and remained at over 15 per week, from week 30 to week 37. The numbers of total WNV infections and WNND cases were also respectively the highest recorded since the emergence of the virus in the country in 2010. Moreover, the 2018 WNND cases accounted for more than a third of the total number of such cases recorded since 2010 (34%, 243/717). This suggests a more intense seasonal virus circulation during 2018. In this regard, large outbreaks occurred in other central European and Mediterranean countries simultaneous to the 2018 WNV seasonal outbreak in Greece [53].

The increased number of cases diagnosed in Greece in 2018 compared with previous years cannot be attributed to a higher sensitivity of the surveillance system, as enhanced surveillance has been consistently implemented in the country during each transmission season since 2010. This has included raising awareness of physicians on an annual basis, publishing updated weekly surveillance reports and providing free-of-charge diagnosis. The large number of WNF cases diagnosed in 2018 (22% of the total symptomatic cases), and in previous years (Table 1), further indicates the enhanced awareness of physicians.

In 2018, the case definition was expanded to include asymptomatic cases detected during blood screening in affected areas; however, this change did not crucially affect the overall amount of recorded WNV infections, given the small number of diagnosed asymptomatic cases (n = 6). Either way, the number of WNND cases (which describes the burden more accurately and is usually preferred for comparisons) was also the largest ever recorded in Greece.

The higher case fatality for WNND cases in Greece compared with that in other European countries [9] could be at least partly attributed to the fact that the Greek surveillance system records all fatalities during the whole hospitalisation period of the patients, which may last for several months (as cases are actively followed up on a daily basis). Furthermore, all deaths of patients with WNV infection are included in the count of fatalities even when they are not directly attributed to WNV infection.

Climatic conditions (mild winter, high temperatures in spring and early summer, as well as rainfall with flooding in the summer), may have contributed to an early and intense WNV circulation in 2018 in Europe [53,54]. The year 2018 was one of the warmest years on record for Europe, with a period of particularly warm weather beginning in late spring, and summer. In most of the years of the 21th century, the average temperatures remained higher than the 1981–2010 average, and years with a long duration of positive anomalies were not unusual; however, 2018 was a notable year with much larger anomalies. In addition, the southeast of Europe experienced an unseasonably wet summer in 2018, ranking among the sixth wettest since 1950 [55]. According to the Hellenic National Meteorological Service, above average temperatures were also recorded in mainland Greece during spring 2018, with the hottest April ever recorded since 1960 in many areas of the country. Temperatures in May 2018 were also higher than the 1971–2000 average in WNV affected areas. In June and July 2018, unusually high precipitations were recorded, with a rainfall increase of more than 200% above the normal values (1971–2000), almost nationwide, which resulted in flooding in several areas, including areas of both Central Macedonia and Attica regions (the 2018 WNV epicentres) [56].

In terms of geographical spread, the first WNV infection outbreak in Greece in 2010 was recorded in the region of Central Macedonia, northern Greece [57]. In the following years, the virus spread further north-east as well as southwards, affecting also the Attica Region. In 2014, only a few cases were diagnosed in the whole country, while no case was reported in 2015–2016. Human cases were recorded again in 2017, limited to a new geographical area, in Peloponnese, southern Greece. The limited number/absence of cases recorded in some seasons in previously highly affected areas might be partially attributed to acquired long-term immunity of local bird populations which diminished the virus circulation [58,59].

In 2018, the geographical distribution of human cases was widely spread, with more areas affected compared with previous years, indicating a nationwide circulation from south to north. The two main epicentres were Central Macedonia, the epicentre of the first recorded outbreak in Greece in 2010, and Attica, an epicentre of the 2011–2013 seasonal outbreaks. In the 2018 season, both rural and urban areas were affected, including large cities, in contrast to the first WNV outbreaks in Greece, which occurred mainly in rural areas.

WNV lineage 2 of the Central European/Hungarian subclade, which is observed in Greece since 2010, was repeatedly detected in all regions, suggesting the establishment of this lineage in Greece. A recent study based on evolutionary dynamics of lineage 2 WNV based on whole genome sequences showed that three novel, independent introductions from Hungary and Bulgaria caused the 2018 seasonal outbreak of WNV in northern Greece [60]. It is of interest that a strain of the Eastern European/Russian subclade of WNV lineage 2 was detected in a case in north-eastern Greece, indicating a new strain introduction. This was not unexpected, given the geographical location of Greece at the crossroads of three continents and the plethora of wetlands in the specific area of northern Greece, where a wide variety of migratory birds stop, with the potential to spread pathogens – while infectious – at the local level.

WNV has become endemic in Greece, as in several neighbouring and other European countries [8,9]. Despite the large number of cases diagnosed on a daily basis, enhanced surveillance of human cases was continuously performed with a timely case investigation and identification of the probable place of infection. The established enhanced surveillance system, and the timely communication and awareness of clinicians and public health professionals, combined with the high level of laboratory response, are vital for the early detection of human cases and the prompt implementation of response measures (vector control, communication, blood safety measures). Timeliness is of major importance, as the early detection of cases and identification of the affected areas is crucial for reducing the spread of WNV and other VBD.

Vector and animal surveillance are also considered useful for the early identification and the geographical characterisation of the WNV circulation, in order to guide response measures [31,61]. According to the national programme for the WNV surveillance in animals in Greece, WNV surveillance in equids and wild birds is planned, organised and coordinated during the transmission season, on an annual basis by the national animal health authorities. Information is shared on a daily basis between public health and animal health national authorities.

Following the severe WNV transmission season in 2018, an After Action Review was performed in Greece (as well as in three other countries, Italy, Serbia and Slovenia) organised by the European Centre for Disease Prevention and Control. This included stakeholders from national, regional and local levels, in order to identify best practices, lessons learned, preparedness gaps and areas for improvement [62]. Enhanced surveillance and diagnostic capacity, strong commitment of involved stakeholders and inter-sectoral collaboration in the context of One Health (national committees, working groups) were identified as strong points. Resources, jurisdictions, procurement design and One Health coordination strategy were identified as areas needing further improvement. The evaluation of effectiveness and impact of vector control measures and biocides use was also highlighted as a priority area to guide targeted interventions and improve preparedness and response.
